# High Risk of Early Cataracts in Young Type 1 Diabetes Group: A Nationwide Cohort Study

**DOI:** 10.1155/2020/8160256

**Published:** 2020-10-09

**Authors:** Wen-Li Lu, Po-Chih Shen, Chen-Hao Lee, Yu-Tsun Su, Li-Min Chen

**Affiliations:** ^1^Division of Genetics and Metabolism, Children's Hospital of China Medical University, Taichung, Taiwan; ^2^Department of Orthopaedic Surgery, Kaohsiung Medical University Hospital, Kaohsiung, Taiwan; ^3^Graduate Institute of Medicine, College of Medicine, Kaohsiung Medical University, Kaohsiung, Taiwan; ^4^Department of Pediatrics, E-Da Hospital, Kaohsiung, Taiwan; ^5^School of Medicine, I-Shou University, Kaohsiung, Taiwan

## Abstract

**Background:**

Chronic hyperglycemia in type 1 diabetes (T1D) patients results in ocular problems over time, but only a few studies emphasized on cataracts.

**Aim:**

To evaluate the epidemiology of cataracts in the T1D population.

**Method:**

A two-part study was conducted using data from the National Health Insurance Research Database in Taiwan. Information from the Longitudinal Health Insurance Database (LHID) was served as a template of the general population. In the first part, a total of 3,622 T1D cases registered between 1998 and 2007 were enrolled and compared with a matched group from the LHID. For identifying risk factors of cataracts in the T1D population in the second part, a total of 9032 T1D cases registered between 1998 and 2013 were included.

**Result:**

Compared to the LHID, the hazard ratio (HR) of cataracts in the T1D group was 5.81 (95% CI 4.60–7.33), and the HR was higher in females (6.29, 95% CI 4.63–8.55). The peak incidence of cataracts occurred between age 20 and 29 in the T1D group, while in the LHID, it was after 60. The overall incidence of cataracts in the T1D group was 9.1%. In T1D patients with cataracts, they were found with higher rates of associated diabetic complications.

**Conclusion:**

Compared to the nondiabetic population, cataracts seemed more rampant and premature in T1D patients, especially those of female gender. Early ophthalmologic examination should be considered in T1D patients.

## 1. Introduction

Diabetes mellitus is a chronic condition requiring lifelong medical care with multidisciplinary managements beyond glycemic control [1]. Chronic hyperglycemia results in ocular problems over time, and most care was emphasized on retinopathy [2, 3]. Diabetes, however, is also a well-known risk factor for the development of cataracts [4, 5], which are one of the significant causes of visual impairment. The number of cataract-related blindness worldwide went from 12.3 million in 1990 to 20 million in 2010 [6, 7]. The main cause of cataracts is aging, but various etiologies also take their parts, including metabolic diseases, radiation, congenital disorders, drug-induced changes, and trauma [8–11]. Cataracts are seldom seen in people under the age of 40 [12]. On account of its rising incidence in those after 60 years of age, cataracts are customarily associated with type 2 diabetes as opposed to type 1 diabetes (T1D) [2, 13]. However, it is not uncommon for clinicians to diagnose cataracts in young T1D patients, among some even preceding their T1D presentations [14–17]. The prevalence varies greatly between 0.7% and 29% [18, 19]. According to the Wisconsin Epidemiologic Study of Diabetic Retinopathy, the 10‐year cumulative incidence of cataract surgery was 8% in young diabetic patients [20].

Acute‐onset cataracts may be reversible in young people with timely glucose control [21]. To date, there have been only a few clinical studies published regarding the characteristics of early diabetic cataracts in the young group. This study endeavors to illustrate the epidemiology of cataracts in the T1D population.

## 2. Methods

### 2.1. Data Source

National health insurance (NHI) in Taiwan is a compulsory national program launched in 1995, with over 99% of Taiwan's population enrolled [22]. The National Health Insurance Research Database (NHIRD), derived from claims data of NHI, can thus illuminate the disease burden and health care process of the entire Taiwanese population [23]. The NHIRD provides a nationwide scale for a retrospective cohort study. The NHIRD deidentifies all registered individuals; thus, no informed consent was obtained in this study. Several data subsets are constructed based on the registration files and original claim data in the NHIRD for research purposes [24]. Considering the lifelong medical demand and possible complications, T1D was classified as a catastrophic illness in the NHIRD [25]. Catastrophic illness dataset, a subset of the NHIRD, comprises medical records of patients with certain severe diseases. Under the NHI program, patients with some certain severe illnesses can apply for catastrophic illness certification, so as to be waived from certain payments for each healthcare encounter [26]. Only those undergone expert verification would be subsumed, and therefore, the diagnosis were considered highly accurate [23]. To increase the validity of the T1D diagnosis, only subjects registered in the catastrophic illness dataset were included in this study.

This study also used information from the Taiwan Longitudinal Health Insurance Database (LHID) 2000. The LHID 2000 is also a subset of the NHIRD. It comprised of the complete claims data of 1,000,000 individuals, randomly sampled from the Registry for Beneficiaries (*n* = 23.75 million) in 2000 and includes data since 1998–2013. The LHID, verified to have no significant difference from the original NHIRD in gender, age (stratified every five years), generation, and categories of insurance premium, was used to be served as a model for the general population in Taiwan [24, 27]. Diagnostic codes used in the NHIRD were based on the International Classification of Diseases, Ninth Revision (ICD-9). This study was approved by the Room for Database Research, E-DA HEALTHCARE GROUP (No. EDAD10603).

### 2.2. Study Population

A two-part study was conducted, and T1D patients registered in the catastrophic illness dataset were enrolled.

To compare with the LHID, T1D patients without preexisting cataracts (ICD-9-CM codes 366.xx) newly registered in the catastrophic illness dataset between 1998 and 2007 were included to ensure a minimum of five years of comparison. To reduce the confounding effect of radiation exposure, patients undergone computed tomography (CT) scans on any part of the body (procedure code: 33070B nonenhanced, 33071B enhanced, and 33072B enhanced and nonenhanced) before the diagnoses of cataracts were excluded. Individuals were selected from the LHID by matching generation, age, and gender to the target T1D group, and those with prior cataracts or CT scans were also excluded. Previous steroid uses include systemic use of beclomethasone, budesonide, mometasone, triamcinolone, methylprednisolone, and prednisolone in any outpatient or impatient visit. The endpoint of the study was the first occurrence of cataract. Subjects who had at least one hospitalization or two clinical visits coded as ICD-9-CM 366.xx within one year were identified as having cataracts.

The second part of the study included all newly registered T1D patients during 1998–2013. Patients with previous cataracts and CT scans of any sites were excluded. Patients who had at least one hospitalization or two clinical visits coded as ICD-9-CM 362.xx within one year were identified as having retinopathy. Patients who underwent amputation of any reasons were identified as procedure codes 64022B, 64023B, 64024B, 64025B, and 64025C in the NHIRD. End-stage renal disease (ESRD) was also recognized as catastrophic illnesses by the Taiwan NHI, and only patients with ESRD registered in the catastrophic illness dataset would be included. Previous steroid uses were as above described.

### 2.3. Statistics

All data were expressed as frequency (percentage) or mean (±standard deviation). Parametric continuous data were compared by the unpaired Student's *t*-test. Freedom from cataract was assessed using Kaplan–Meier analysis, and the significance was determined using the log-rank test. The disease-free time was calculated from the date of enrollment to the date of first diagnosis of cataracts. All *p* values less than 0.05 were considered significant. All data management and calculations of hazard ratios (HRs) were carried out with SAS version 9.4 (SAS Institute, Cary, NC, USA).

## 3. Result

**Figure 1:**
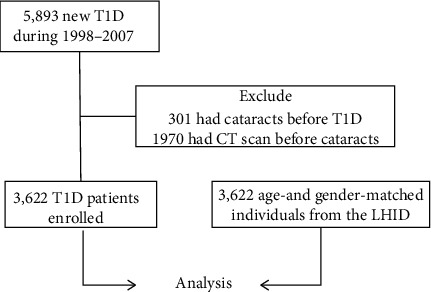
Study flow chart. Between 1998 and 2007, 5,893 patients were identified with new-onset type 1 diabetes (T1D) in Taiwan. After excluding 301 of those with pre-T1D cataracts and 1,970 of which computed tomography (CT) imaging studies were performed prior to being diagnosed with cataracts, a total of 3,622 cases were included. Equal numbers of age- and gender-matched individuals were selected from the Longitudinal Health Insurance Database (LHID) for analysis.

Between 1998 and 2007, 5,893 patients were identified with new-onset T1D in Taiwan. After excluding 301 of those with pre-T1D cataract and 1,970 of which CT imaging studies were performed prior to being diagnosed with cataract, a total of 3,622 cases were included for analysis (Figure 1). Matched individuals selected from the LHID showed no significant difference from the target T1D groups in terms of age and gender (Table 1). Percentage of previous steroid use was also listed in the table, and the T1D group had a lower rate than the LHID counterpart.

**Figure 2:**
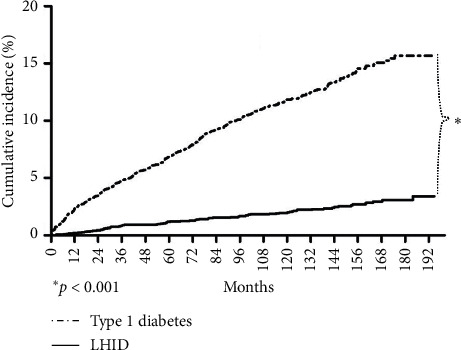
The cumulative incidence of cataracts in the type 1 diabetes group vs. the Longitudinal Health Insurance Database (LHID) group.

**Figure 3:**
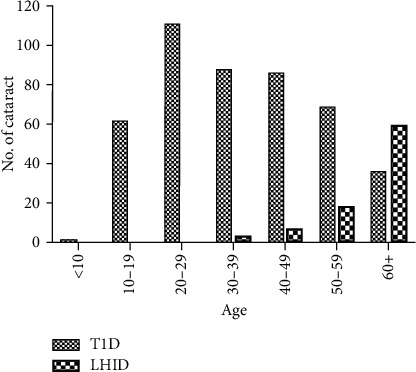
Age of cataracts onset in the type 1 diabetes (T1D) group vs. the Longitudinal Health Insurance Database (LHID) group.

The hazard ratio of cataracts between the T1D group and the LHID group stratified by age of enrollment and gender is shown in Table 2. The overall hazard ratio was 5.81 (95% confidence interval (CI): 4.6, 7.33) for the T1D group. It was higher for all age except after 60 and highest at young age, up to 112.6 (95% CI: 15.72–807.2) in age 20–29. Females had a higher HR (6.29, 95% CI: 4.63–8.55) than males (5.20, 95% CI: 3.64, 4.75). The cumulative incidence of cataracts in the T1D group and the LHID group is shown in Figure 2. The incidence of cataracts was higher in the T1D group immediately after enrollment (*p* < 0.001). The age of cataracts onset is displayed in Figure 3. In the LHID group, the cataracts were rarely seen before age 40, and the incidence gradually increased as aging. On the other hand, the incidence of cataracts in the T1D group peaked in their twenties and then fell gradually in the subsequent decades.

For the second part of the study, a total of 9,503 T1D cases were registered between 1998 and 2013. After excluding 471 cases with pre-T1D cataracts, 9,032 cases were enrolled. The demographic features of T1D patients are revealed in Table 3. The mean age of T1D diagnosis was 23 ± 14.75 years, while half of the patients had T1D diagnosis before their twenties. 4,367 (48.4%) of the patients were male.

**Figure 4:**
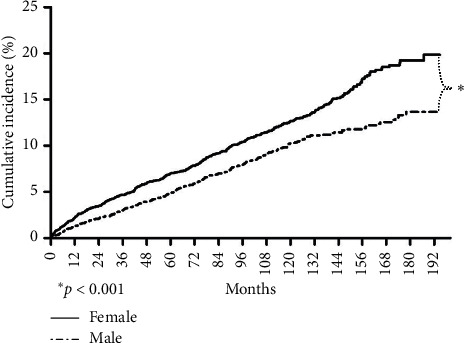
The cumulative incidence of cataracts in the type 1 diabetes group divided by gender.

Totally, 820 (9.1%) patients were diagnosed with cataracts. Based on the absence or presence of cataract, the comparison of distribution of associated diabetic comorbidity is demonstrated in Table 4. Patients with cataracts had a higher rate of experiencing retinopathy, amputation, and ESRD, all with a significant *p* value.  Figure 4 demonstrated the cumulative incidence according to gender. Females had significantly higher incidence than males (*p* < 0.05).

## 4. Discussion

Most ophthalmologic emphasis of T1D has been stressed on the influence of retinopathy [2, 28], and the lesser known is the complication of cataract, which has resulted in more than half of the blindness worldwide [12]. Untreated cataracts in children cast tremendous burden to the child and family [29]. Our study revealed that T1D patients certainly had a higher risk of developing cataracts than nondiabetic population. Because retinopathy is considered to take at least 5 years to develop after the onset of hyperglycemia, the latest guideline of American Diabetes Association suggests eye examination 3–5 years after diabetic onset [2, 28]. However, nearly all articles addressing diabetic cataracts deemed that it might tend to occur early in the course of T1D [14–18, 30–32]. In our study, the hazard ratio of diabetic cataracts was tremendously high in the young group and then declined gradually as senile cataracts developed in the general population. The peak incidence of new-onset cataracts in the T1D group occurred between age 20 and 29 and then declined gradually, a trend that contradicts that of the LHID. In the LHID group, cataracts were rarely seen before age 40, and that was compatible to common global status [33, 34]. This might be the first article to provide a clear picture of the early tendency of diabetic cataracts in T1D patients. Besides, 301 patients with pre-T1D cataracts were excluded in our study to minimize the confounding effect of other possible cataractogenic factors. The high percentage (5.1%) of pre-T1D cataracts seemed to concur with the inclination. We highlight the substantial gap in the epidemiological knowledge of diabetic cataracts. Pediatric cataract can have a better prognosis if diagnosed and managed early. Surgery, the gold standard of treatment, saves vision efficaciously [35]. It is not without complication, however. The long-term effect of diabetes and the fact of growing of anterior eye segment in children make it thornier [36, 37]. Early cataracts might be reversible without surgery [21, 38]. Transient cataracts during the course of diabetic ketoacidosis have been reported in children [39, 40]. We suggest lens examination at the beginning of T1D, and that was also warranted by some experts and the International Society for Pediatric and Adolescent Diabetes [3, 18, 30].

Why some children with T1D are more predisposed to develop cataracts that remains unclear. The aldose reductase pathway is widely accepted as implicated in the pathogenesis of diabetic cataracts based on findings in animal models [5, 41]. Aldose reductase, an enzyme primarily residing in the lens epithelium, converts excess glucose to sorbitol when the blood glucose level is elevated [42, 43]. Sorbitol penetrates cell membranes poorly, accumulates in the lens, increases the osmotic pressure, and leads to lens vacuolization, lamellar separation, crystalline fiber damage, and ultimately cataract formation [43, 44]. The early tendency of diabetic cataracts indicates that hyperglycemia alone cannot be responsible for the pathogenesis. Certain predisposing factors might exist and contribute to the rapid development. Other plausible explanations include oxidative stress, osmotic damage, glutathione loss, decreased adenosine triphosphate production, and autoimmunity [45–47]. Given that most hypotheses require a period of time for cataracts to develop, they could not fully explain why some patients developed cataracts shortly after contracting T1D. Genetic predisposition was suggested, and some candidate genes pertaining to diabetic cataract have been found [14, 32].

Most studies found that women exhibited a higher rate of cataracts than men [12, 48]. In view of diabetic cataracts, female preponderance still existed [30, 32, 49, 50]. Our study also revealed such a trend. It has been suggested that increased levels of aldose reductase in females might contribute to the higher prevalence [14], but the real mechanism is still not fully understood.

A risk of bias exist in the fact that the T1D patients are likely to have more outpatient and inpatient utilization than the general population, which might result in faster detection of small, nondeteriorating vision changes and more diagnosis of cataracts. The hazard ratio might thus be overestimated, but that could not explain the decreasing trend with aging. If the high hazard ratio was totally ascribed to such bias, we should expect a steadily increasing HR over time. Of note, as cataract is a multifactorial disease, bias might also be a concern in other confounding factors. To minimize such bias, not only were those with pre-T1DM cataracts excluded in this study but also those who received CT imaging studies prior to being diagnosed with cataracts. Theoretically, only CT scans of the head and neck would increase the risk of radiation-induced cataracts. However, selecting specific sites of CT scans was hampered because the procedure codes of CT scans of any sites in the NHIRD were all the same; thus, all precataract CT scans were excluded in this study regardless of the imaging sites. On the other hand, previous steroid use, also a well-known risk factor of cataracts [10], was not excluded in this study or the study group and might be too small to have statistic power. Nonetheless, owing to the lower rate of steroid use in the T1D group of the first part and in the cataracts group of the second part, causes to be clarified, we deemed that it played a minimal role in diabetic cataracts. In addition, poor glycemic control was implicated in diabetic cataracts in many studies [17, 18, 30, 51]. Hemoglobin A1c level was not registered in the NHIRD; thus, we tried to probe glycemic control by other vascular complications. Irreversible solid outcome, amputation, and end-stage renal disease were selected to minimize possible registrations errors. Higher rate of these diabetic complications in the cataract group seemed implying association between poor sugar control and cataract formation.

## 5. Conclusion

Compared to the age- and gender-matched general population, T1D patients were at a higher risk for cataracts. The overall hazard ratio was 5.81 (5.20 in males and 6.29 in females), and the ratio was even more prominent, >112.6, before age 30. Considering the early tendency of diabetic cataracts in T1D patients, ophthalmologic examination should be considered at the onset of diabetes in all children.

## Figures and Tables

**Table 1:** The demographic features of the type 1 diabetes (T1D) group and the matched group selected from the Longitudinal Health Insurance Database (LHID). The distribution of age and gender was similar between the two groups.

	T1D	LHID	*p* value
Age, yrs (mean ± SD)	20.33 ± 13.86	20.74 ± 14.21	0.214
<10(%)	918 (25.4)	918 (25.4)	1.000
10–19(%)	1169 (32.3)	1169 (32.3)	
20–29(%)	717 (19.8)	717 (19.8)	
30–39(%)	476 (13.1)	476 (13.1)	
40–49(%)	220 (6.1)	220 (6.1)	
50–59(%)	74 (2.0)	74 (2.0)	
60+(%)	48 (1.3)	48 (1.3)	
Male(%)	1752 (48.4)	1752 (48.4)	1.000
Steroid use(%)	770 (21.3)	1035 (28.6)	<0.001
Total	3622	3622	

**Table 2:** The hazard ratio (HR) of cataracts between the type 1 diabetes (T1D) group and the Longitudinal Health Insurance Database (LHID) group stratified by age of enrollment and gender. The younger T1D group had a higher HR of cataracts.

	T1D (*n* = 3622)		LHID (*n* = 3622)		Crude HR (95% CI)
	Cataract no(%)	Time to cataract, mo±SD	Cataract no(%)	Time to cataract, mo±SD	
Follow-up (mo)	120.20 ± 42.22		133.02 ± 35.60		
Age of enrollment (yrs)					
<10	17 (0.5)	73.51 ± 38.48	0 (0.0)	N/A	N/A
10–19	101 (2.8)	62.96 ± 47.12	0 (0.0)	N/A	N/A
20–29	101 (2.8)	52.36 ± 40.41	1 (0.02)	124.83 ± 0.00	112.6 (15.72, 807.2)^*∗*^
30–39	94 (2.60)	64.62 ± 47.38	8 (0.2)	119.26 ± 58.73	13.75 (6.68, 28.31)^*∗*^
40–49	69 (1.91)	61.29 ± 39.43	14 (0.4)	78.25 ± 45.89	6.10 (3.43, 10.85)^*∗*^
50–59	40 (1.10)	51.05 ± 34.85	29 (0.8)	77.62 ± 43.55	1.79 (1.11, 2,89)^*∗*^
60+	25 (0.69)	22.01 ± 25.34	32 (0.9)	44.34 ± 43.72	0.86 (0.51, 1.45)
Gender					
Male	176 (4.9)	56.49 ± 39.24	36 (1.0)	56.03 ± 38.28	5.20 (3.64, 7.45)^*∗*^
Female	272 (7.5)	58.47 ± 45.74	48 (1.3)	79.74 ± 51.62	6.29 (4.63, 8.55)^*∗*^
Total	448 (12.4)	57.69 ± 43.27	84 (2.3)	69.58 ± 47.61	5.81 (4.60, 7.33)^*∗*^

^*∗*^ *p* < 0.001.

**Table 3:** The demographic features of type 1 diabetes (T1D) patients newly registered during 1998–2013.

Age of diabetes onset (yrs)	*n* (%)
Mean ± SD	22.99 ± 14.75
<10	1769 (19.6)
10–19	2754 (30.5)
20–29	1859 (20.6)
30–39	1432 (15.8)
40–49	772 (8.6)
50–59	281 (3.1)
60+	172 (1.9)
Male	4367 (48.4)
Total	9032

**Table 4:** The distribution of associated factors according the presence of cataracts.

	Cataract		*p* value
	Absent (*n* = 8212)	Present (*n* = 820)	
Male	4030 (49.1)	337 (41.1)	<0.001^*∗*^
Retinopathy	1021 (12.4)	314 (38.3)	<0.001^*∗*^
Amputation	44 (0.5)	12 (1.5)	0.001^*∗*^
ESRD	136 (1.7)	61 (7.4)	<0.001^*∗*^
Steroid	1926 (23.5)	98 (12.0)	<0.001^*∗*^

ESRD, end-stage renal disease.

## Data Availability

The data used to support this study are available from the National Health Insurance Research Database (NHIRD) published by Taiwan National Health Insurance (NHI) Bureau. Due to legal restrictions imposed by the government of Taiwan in relation to the “Personal Information Protection Act,” data cannot be made publicly available. Requests for data can be sent as a formal proposal to the NHIRD (http://nhird.nhri.org.tw).
